# Expression profiling and functional annotation of noncoding genes across 11 distinct organs in rat development

**DOI:** 10.1038/srep38575

**Published:** 2016-12-09

**Authors:** Zhuo Wen, Geng Chen, Sibo Zhu, Jinhang Zhu, Bin Li, Yunjie Song, Suqing Li, Leming Shi, Yuanting Zheng, Menglong Li

**Affiliations:** 1 College of Chemistry, Sichuan University, Chengdu 610064, China; 2 Center for Pharmacogenomics, School of Pharmacy, and State Key Laboratory of Genetic Engineering and MOE Key Laboratory of Contemporary Anthropology, School of Life Sciences, Fudan University, Shanghai 201203, China; 3 Collaborative Innovation Center for Genetics and Development, Fudan University, Shanghai 200438, China

## Abstract

Accumulating evidence suggests that noncoding RNAs (ncRNAs) have important regulatory functions. However, lacking of functional annotations for ncRNAs hampered us from carrying out the subsequent functional or predictive research. Here we dissected the expression profiles of 3,458 rat noncoding genes using rat bodymap RNA-sequencing data consisting of 11 solid organs over four developmental stages (juvenile, adolescent, adult and aged) from both sexes, and conducted a comprehensive analysis of differentially expressed noncoding genes (DEnGs) between various conditions. We then constructed a co-expression network between protein-coding and noncoding genes to infer biological functions of noncoding genes. Modules of interest were linked to online databases including DAVID for functional annotation and pathway analysis. Our results indicated that noncoding genes are functionally enriched through pathways similar to those of protein-coding genes. Terms about development of the immune system were enriched with genes from age-related modules, whereas terms about sexual reproduction were enriched with genes in sex-related modules. We also built connection networks on some significant modules to visualize the interactions and regulatory relationship between protein-coding and noncoding genes. Our study could improve our understanding and facilitate a deeper investigation on organ/age/sex-related regulatory events of noncoding genes, which may lead to a superior preclinical model for drug development and translational medicine.

Functional investigations on protein-coding genes have been well studied, whereas the functions of noncoding RNAs (ncRNAs) are rarely understood. Previously, transcribed ncRNAs were simply regarded as accumulated debris from intronic and intergenic regions of the genome during the transcriptional process^1^,^2^. However, ncRNAs play key roles in the regulation of many biological, pathological and developmental processes. For example, ncRNAs can regulate the translation of messenger RNAs (mRNAs) to proteins and play a determinant role in cellular behaviors^3^,^4^,^5^. To date, the exact functions of most ncRNAs remain largely unclear to us^6^, and even their expression profiles are not well explored. The well-known ncRNAs, ribosome RNAs (rRNAs), were reported to be involved in mRNA transcription, RNA silencing and post-transcriptional regulation of gene expression, and even relevant to cancer metastasis^7^. Moreover, pseudogenes can potentially go through the process of transcription, and small nucleolar RNAs (snoRNAs) can exhibit tissue-specific and developmental regulation or imprinting, indicating various regulatory functions of ncRNAs^8^,^9^,^10^,^11^,^12^,^13^,^14^. Thus, more attention should be paid to these poorly understood but crucial regulators, ncRNAs.

Previous studies indicated that noncoding genes show more sensitivity to organ and age factors compared to protein-coding genes, thus we chose ten different kinds of solid organs from each rat across four ages to study the regulatory changes in rat development^15^,^16^ by using RNA-seq, a revolutionary technology based on next- generation sequencing for new discoveries from transcriptomes^17^. RNA-seq can generate a snapshot of the identity and quantity of all kinds of RNAs transcribed from a genome at a given time and can provide us with a clearer understanding of the expression profiles of genes under different conditions.

Rat is the second most widely used model animal for biomedical research. It has greatly contributed to our understandings of the mechanisms of diseases and has served as a great tool for safety evaluation of numerous medicines^18^. The FANTOM consortium published a set of long noncoding RNAs (lncRNAs) in mouse and mammalian by cDNA sequencing^19^,^20^,^21^. GENCODE announced a catalog of human intergenic lncRNAs based on computational predictions and annotation using RNA-seq^3^. The mouse ENCODE consortium has also made progress in identifying tissue-specific regulatory elements in mouse^22^,^23^. However, few studies reported the developmental effects of lncRNAs on mouse brain^24^, liver^15^ and heart^10^. Furthermore, many databases like Rfam, NONCODE, LNCipedia, and LncRNADisease are consistently being updated in their resources^25^,^26^,^27^,^28^. Thus, a comprehensive characterization and annotation of the noncoding genes in different organs across distinct developmental stages of rat is very important to the research community.

Here we characterized DEnGs from multiple perspectives on 3,458 noncoding genes among 320 rat RNA-seq samples covering 11 solid organs across four developmental stages for both female and male. We also constructed the co-expression network between noncoding genes and protein-coding genes. Some significant and meaningful modules related to organ, age and sex were revealed. We further annotated these related modules to explore the potential functions of ncRNAs. Our study provides a comprehensive overview of the expression characteristics of ncRNAs in rat.

## Results

### Rat noncoding genes were expressed at lower levels but are more organ specific

**Figure 1:**
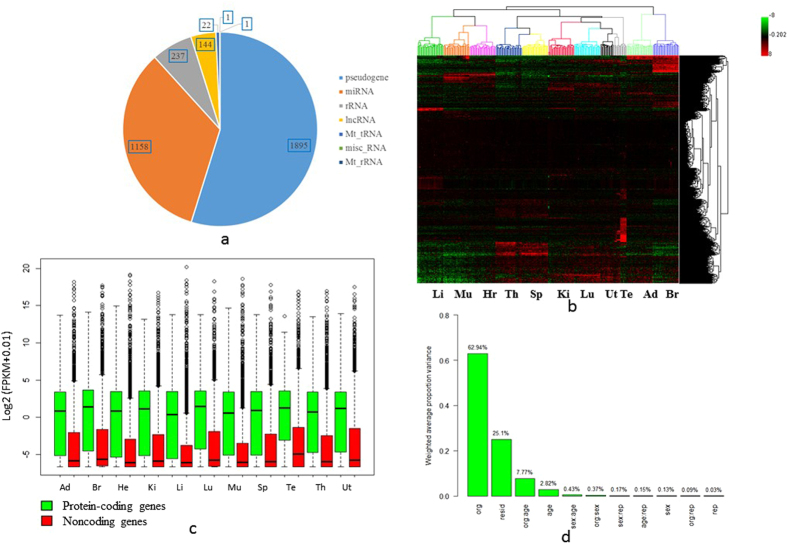
Expression profiling of noncoding genes. (**a**) Type classification of the 3,458 expressed noncoding genes used in our study based on Ensembl Rnor_5.0. (**b**) Hierarchical clustering analysis of 320 rat samples based on gene-expression profiles of 3,458 noncoding genes. (**c**) Expression level of coding genes and noncoding genes in terms of log 2 (FPKM + 0.01). (**d**) Principal variance component analysis (PVCA) of the relative importance of the contributing factors (organ, age, sex and replicate) and their combinations to the total variance in expression profiles. Organs tested are: Ad, adrenal; Br, brain; Hr, heart; Ki, kidney; Li, liver; Lu, lung; Mu, skeletal muscle; Sp, spleen; Te, testis; Th, thymus; and Ut, uterus.

To obtain an overview of the expression profiles of noncoding genes in rat, we first extracted the expression values of 3,458 noncoding genes with expression level FPKM > 0.01 using Cufflinks^29^. These 3,458 noncoding genes were divided into seven subtypes including pseudogene, miRNA, rRNA, lncRNA, mitochondrial tRNA and rRNA, and miscellaneous. About 54.8% (1,895) are pseudogenes, 33.5% (1,158) are miRNAs, and 6.8% (237) are lncRNAs (Fig. 1a). We then performed hierarchical clustering analysis (Fig. 1b) of the expression profiles of 320 rat samples. The dataset includes ten organs per rat (i.e. adrenal gland, brain, heart, kidney, liver, lung, muscle, spleen, thymus and testis for male or uterus for female), with age of 2-weeks old (juvenile), 6-weeks old (adolescence), 21-weeks old (adult) and 104-weeks old (aged). As expected, the expression profiles of noncoding genes were clearly clustered by organ type and showed more organ specificity than coding genes as observed previously^30^. The grouping of different organ types reflected their respective biological characteristics. For example, muscle and heart were clustered side by side, and it can be explained by the fact that both muscle and heart consist of smooth muscle tissue. Consistent with previous studies, the overall expression level of ncRNAs is lower than that of coding genes across all 11 tissues included in this study (Fig. 1c).

### Age contributed more than sex to the overall variance in expression profiles of nocoding genes

From the principal variance component analysis (PVCA) (Fig. 1d), we found that organ contributed the most (62.94%) among all the sources of variance in our data set, followed by residues variance (25.10%). Age (2.82%) contributed more than sex (0.13%) for the overall variance in expression profiles of noncoding genes, compared with protein-coding genes^17^. This is different from the trend of protein-coding genes but in accordance with previous reports. Thus, noncoding genes appeared to be critical gene regulators for development. However, the fact that the rat Y chromosome was not well annotated might have also contributed to the relatively small contribution of sex to the overall variance in expression profiles.

### Juvenile and aged rats shared a larger number of age-related DEnGs

**Figure 2:**
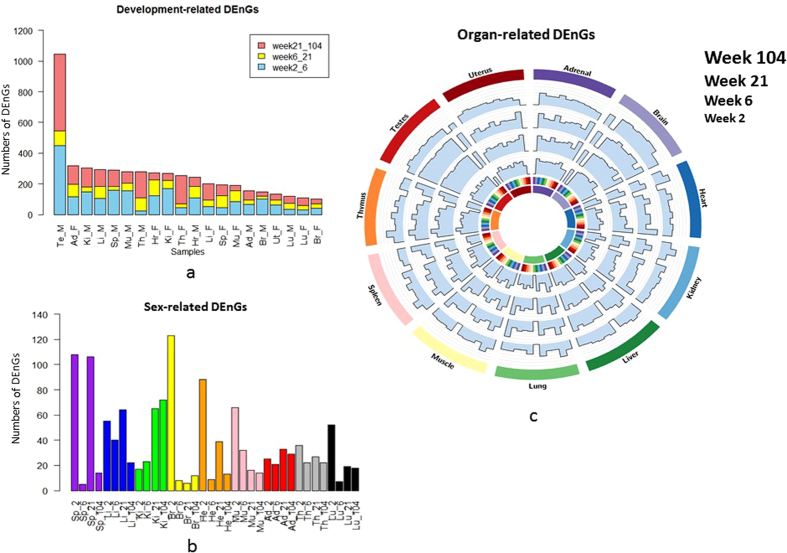
Identification of differentially expressed noncoding genes. (**a**) Development-related differentially expressed noncoding genes. (**b**) Numbers of sex-specific differentially expressed noncoding genes (DEnGs). The DEnGs from 288 samples (exclude uterus and testis samples) were generated by Cuffdiff based on Benjamini-Hochberg-correlated *P* ≤ 0.05, across four developmental stages and nine organs. (**c**) Organ-specific differentially expressed noncoding genes among samples of both sexes.

To explore the development-related DEnGs, we used Cuffdiff for all genes including noncoding ones at both gene and transcript levels. We performed statistical analysis to identify significant DEnGs between any two consecutive developmental stages across 11 organs, and between sexes. The threshold was set as Benjamini-Hochberg adjusted p-value ≤0.05 plus an expression-level change of at least two times higher or lower (fold change ≥2 or ≤0.5). Generally, male rats shared more developmental stage-dependent DEnGs than female except for adrenal gland and heart. The numbers of DEnGs varied from 19 (brain in female rat) to 503 (testis) across all tissues (Fig. 2a). Adrenal gland, heart, kidney, liver, muscle and thymus exhibited fairly equal numbers of developmental stage-dependent DEnGs, whereas brain and lung showed the least numbers of DEnGs. We speculated that some cells like neurons become mature early in life and will not change easily again afterwards^31^,^32^,^33^, thus the expression levels of the ncRNAs in these cells become relatively stable over age. Moreover, the largest number of DEnGs across age was found from testis. One possible reason is that specific and dramatic gene-expression changes occurred during sexual maturity and functional decline in aged male rats. Overall, most organs showed certain sex-related DEnGs.

### Sex-related differentially expressed noncoding genes mostly occurred in 2-week-old rats

Although sex difference is not as strong as that of organ or age, we set the same threshold of Benjamini-Hochberg adjusted p-value ≤0.05 with the same fold change cutoff as above on the nine non-sex organs from every developmental stage to identify sex-biased noncoding genes (Fig. 2b). The number of sex-related DEnGs depended not only on organ, but also on age. Spleen expressed the largest number of sex-related DEnGs, whereas lung expressed the least. Adrenal gland and thymus showed fairly consistent numbers of sex-related DEnGs during all development age stages. However, most organs expressed a larger number of sex-specific non-coding genes in juvenile rats, while fewer sex-specific non-coding genes were found in adolescence except for kidney and adrenal gland. Interestingly, brain exhibited the largest number of sex-related DEnGs in juvenile, and this finding was consistent with the fact that brain develops at an amazingly fast rate during early development, especially after birth. For example, by the age of 2 weeks old, the brain is about 80% of the adult size^34^.

### Brain and testis expressed larger numbers of noncoding genes among organs

The selection criteria of organ-related DEnGs between each comparison were set similar as above, *i*.*e*. Benjamini-Hochberg adjusted p-value ≤0.05 and a fold change ≥2 between two organs. The numbers of DEnGs turned out to be varied dramatically as different pairs of organs were compared. A relatively larger number of DEnGs was obtained when brain and liver were compared with other organs among all four developmental stages. For brain, its special biological functions required the expression of a large number of unique genes compared with other organs, whereas for liver, the faster metabolism of liver cells may cause the difference. Yet a fewer number was detected when uterus was compared with other organs. However, for testis and thymus, the number of DEnGs was remarkably age dependent. Testis showed the largest numbers of DEnGs at week 6 and week 21 when in comparison to other organs, but the least numbers of DEnGs occurred at week 2 and week 104 (Fig. 2c). Thymus exhibited many organ-related DEnGs in juvenile and adolescent stages, and the number declined at adult. Non-sexual organs showed no significant developmental stage dependence of the numbers of DEnGs when compared to other organs.

### Construction of co-expression network

To annotate the function of noncoding genes of rat, we constructed a co-expression network to characterize functional relations between protein-coding genes and noncoding genes^35^,^36^,^37^. Nodes in the network denote genes, and edges between genes were determined by pairwise Pearson correlation coefficients. Genes highly correlated are clustered in the same module that might be involved in similar biological processes. Candidate genes in the network were determined by analysis of variance (ANOVA) with Benjamin-Hochberg corrected p-value ≤0.05 among all 320 samples. We identified 3,572 age-related protein-coding and noncoding genes, 1,603 sex-related genes, and 16,346 organ-related genes. We examined whether these modules were enriched with gene products in a specific biological process as defined by Gene Ontology (GO). All GO terms statistically significantly enriched with the candidate genes are presented in Supplementary Tables S1–3.

### Organ-related modules largely dictated the functions of different tissue types

**Figure 3:**
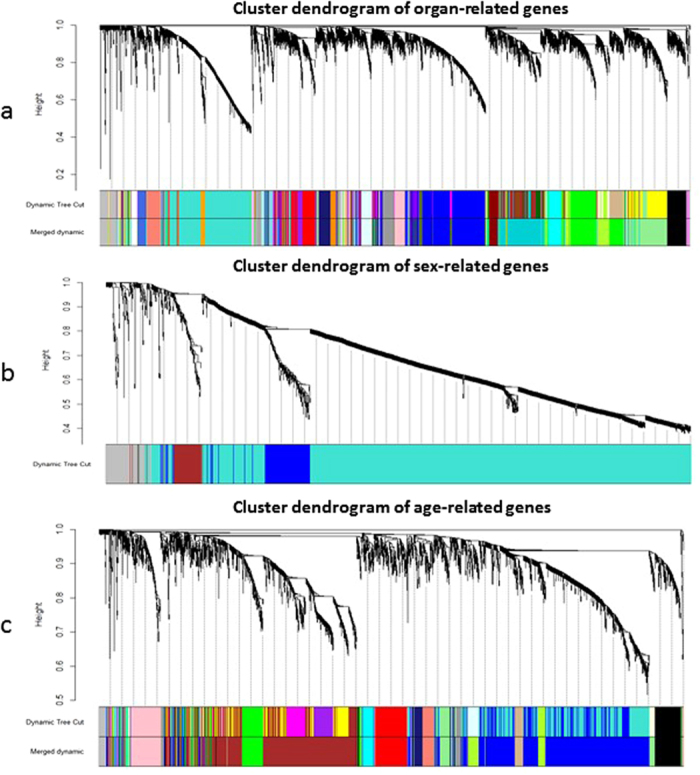
Co-expression network construction on age-, sex-, organ-related genes. (**a**) Clustering dendrogram of sex-related genes, four modules clustered in total after merge. Gene dendrogram obtained by average linkage hierarchical clustering, with dissimilarity based on topological overlap. The color underneath the trees showed the modules assigned after the tree cut. Color grey is reserved for genes outside of all modules. (**b**) Clustering dendrogram of age-related genes, 14 modules clustered in total after merge. (**c**) Clustering dendrogram of organ-related genes, 32 modules clustered in total after merge.

Thirty two (32) distinct gene modules were identified from the 16,346 organ-related genes. The modules were grouped together by similar tissue types or biological systems. First, the blue module (Fig. 3a) consisted of 1,806 GO annotated genes that were remarkably enriched in mental activity related terms like transmission of nerve impulse, synaptic and neuron differentiation, behavioral, learning or memory^38^. In addition, pathways enriched with these genes were also involved in neuron active ligand-receptor interaction and calcium signaling, as well as in long-term depression syndrome. Secondly, the turquoise module (Fig. 3a) consisted of 2,284 genes including 221 noncoding genes, most of which function in sex-related processes, male gamete generation, RNA processing, and ncRNA metabolism, highlighting the important causal relationship between the functions of testis and its rapid energy and gamete metabolism. Thirdly, the big green module (75/1,157) (Fig. 3a) was enriched with genes involved in DNA metabolism, cell cycle, and chromosomal organization^39^,^40^, the basic biological process for the body’s daily bioactivities. Interestingly, this module was also significantly enriched with genes in the p53 signaling pathway. One possible explanation was that cell cycle was closely related to DNA replication, but DNA mismatches without repair could lead to the activation of this signaling pathway^41^. Fourth, the light green module (111/1,289) (Fig. 3a) consisted of genes with functions in cellular respiration, energy and lipid catabolic processes. Pathway analysis results suggested that these complex interactions led to significant enrichment of Parkinson’s disease, Huntingon’s disease, and Alzheimer’s disease. Finally, the black module (Fig. 3a) with 27 noncoding genes were enriched with genes involved in pathways of the arrhythmogenic right ventricular cardiomyopathy, dilated and hypertrophic cardiomyopathy. These results suggested that the black module is remarkably related to heart functionality. The results further verified that the regulatory role of noncoding RNAs is closely associated with human diseases^42^,^43^.

### Sex-related modules involved genes of sexual development

To evaluate the function of sex-related noncoding genes, 1,603 sex-related informative genes were clustered into four modules (blue, brown, turquoise and grey) after dynamic tree cutting (Fig. 3b). Each module contained at least 30 genes and was involved in a sex-related biological process. The turquoise module (Fig. 3b) consisted of 1,260 genes including 23 noncoding genes and represented the biggest group. Among these genes, 535 were significantly enriched in GO biological process terms mostly contributing to testicular functions of male gamete generation, sexual reproduction and spermatogenesis. This finding can explain the larger number of DEnGs in testis and also show that sex differences were mainly reflected in sexual organs. Pathway analysis revealed two significant pathways accounting for basal transcription factors and glycolysis or gluconeogenesis, suggesting that the sex differences could be embodied more in energy production and consumption. Another small-sized brown module (Fig. 3b) contained 86 genes including 20 noncoding genes, which are mainly involved in cell circle and cell circle processes. Those enriched pathways also included oocyte meiosis and cell cycle, which is in line with the above results that these 1,603 sex-related genes were identified as belonging to gene modules associated with sex.

### Age-related gene co-expression modules were relevant to cell division, cell circle process and development of immune system

For the age-related gene co-expression network, 14 stable modules including 3,572 genes were found (Fig. 3c). The blue module (Fig. 3c) consisted of 1,151 genes including 60 noncoding genes that were significantly enriched in GO terms such as cell circle process, organelle fission, mitosis, as well as regulation of transcription and regulation of RNA metabolic process. These terms all indicated that developmental differences are related to metabolic changes and regulations of gene expression during growth and development. Pathway analysis showed significant enrichment of genes involved in cell cycle and base excision repair, suggesting that cell cycle and base self-repair functions might be affected by aging. Other modules like brown (40/875) and green (8/189) (Fig. 3c) tended to show another distinct enrichment trend on regulations of immune system such as immune response, immune system development and lymphoid organ development. Many age-related DEnGs in brain, spleen and thymus are associated with cancer and other aging diseases, suggesting that aging could result in these diseases by affecting the immune system^12^. Other genes mostly function in terms of helping immune system to defeat inflammation and to transfer leucocytes to build the body’s defense system. Finally, seven noncoding genes in the pink module (Fig. 3c) appeared to be associated with skeletal system development and tube development. The results showed that aging can lead to many kinds of changes in the body through changes in gene expression profiles.

### Visualization of age-related brown module showed regulatory role with hub coding genes in the network

**Figure 4:**
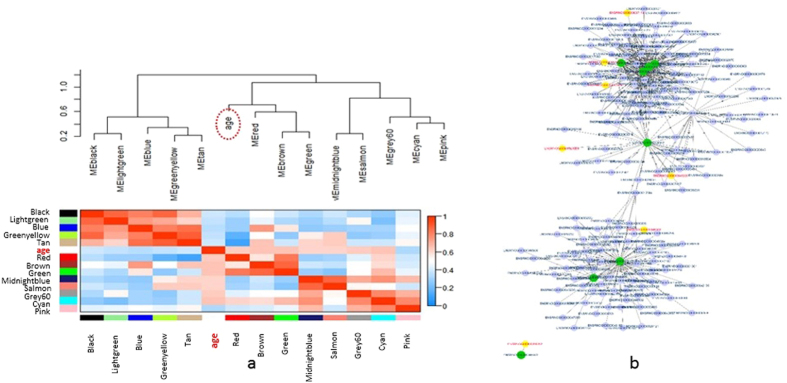
Network visualization of age-related brown module. (**a**) Visualization of the network with 2,000 age-related eigengenes. The upper dendrogram displays the dissimilarity of eigengenes by their correlations. The lower heatmap shows the eigengene adjacency. Red color indicates strong correlation with age and blue stands for weak correlation. (**b**) Visualization of the network connections among the most connected genes in the brown module. The topological overlap for network connections was set above 0.32. Yellow nodes indicate noncoding genes, whereas purple nodes stand for coding genes.

We correlated the eigengenes from 3,572 age-related genes to study the relationships between the identified modules and age (Fig. 4a). As was shown in the plot, the adjacency reflected the correlation between modules. The brown module showed relatively closer relations with aging (Fig. 4a) and contained many noncoding genes, prompting us to use it for the subsequent age-related analysis. Thus, we selected 189 age-related genes (threshold was set as 0.32) including seven noncoding genes from these modules for further network visualization analysis, and the threshold based selection was performed before importing the data for visualization into Cytoscape^44^. We obtained two main clusters with several hub genes in the center connecting the neighbors (Fig. 4b). The brown module was primarily enriched with genes associated with the biological processes of the immune system. For example, the eight main hub coding genes were in charge of the function related to lymphocyte (ENSRNOG00000010319 as lymphocyte cytosolic protein 1, lcp1) or immunoglobulin (ENSRNOG00000029890 as immunoglobulin heavy constant gamma 3, Ighg3). Although noncoding genes were not in the center of this network, seven rat noncoding genes were involved in the network functioning ogether with immune system related genes.

## Discussion

An increasingly large number of publications have emphasized the important regulatory roles of long noncoding RNAs^20^,^45^,^46^,^47^,^48^. However, the expression characteristics and the functional regulation preference of rat noncoding genes were still uncharacterized. Thus we extensively explored different types of rat noncoding genes using the RNA-seq data from 11 solid organs during four key developmental periods in a life cycle, representing the largest catalog of rat noncoding genes to the best of our knowledge. A large number of DEnGs were detected from different perspectives among organs and developmental stages, and organ, age, and sex seem to interact with each other indicating the distribution of DEnGs. For example, the quantity of sex-related DEnGs was related with both organ and age; however, the number of organ-specific DEnGs was seldom affected by age or sex except on sexual organs, like testis. Testis showed more DEnGs from mature rats (at week 6 and week 21) and relatively few at other stages, illustrating its special features for sexual maturity and rapid reproduction. The result was consistent with our previous finding that organ is a more dominant factor contributing to differential expression analysis compared to age and sex.

Our analysis also showed that most noncoding genes were expressed at lower levels than protein-coding genes. Nevertheless, they still play important roles in post-transcriptional regulation, and their functions could in a way be inferred from co-expression network analysis. Here, we built up functional annotations for noncoding genes related with organ, age and sex by linking noncoding genes and protein-coding genes together. In addition, the co-expression network can further bridge the noncoding genes with biological processes. The genes enriched in co-expression network modules were involved in similar cellular compartments due to the reliable modularity^36^. For each module, it could be a consequence of transcriptional regulation by noncoding genes, functioning co-repression or co-activation with coding genes, or coordinately coping with those similarly expressed genes to fulfill the biological functions.

We also found that the tissue specificity is likely driven by the synergistic effect of multi-functional genes. As different types of cells have different ways of metabolism and cell renewal cycle, they may express differently during various developmental periods. For instance, cells in liver and muscle tissues refresh themselves every several months. In general, the number of DEnGs between 6- and 21-week old was lower than those in any other developmental stages, indicating the maturity of the organs at 6-week. Week 2 and week 6 rats displayed the largest number of DEnGs, which could be attributed to puberty. For old rats (104-week), organs like spleen, testis and thymus begin to experience functional recession or atrophy, which also resulted in a large number of DEnGs compared to a previous development stage (21-week). This was also observed by co-expression analysis in that 32 modules were obtained after merging for organ-related genes, of which the number is far bigger than that of organs. That is, genes in modules perform various fundamental cellular functions such as stress response and cell motility from the nerve system. The modules are clustered by genes performing fundamental functionalities of the nerve and neuron systems. The large size of the module is consistent with the complicated biological neural networks present almost everywhere in the human body.

In this study, we provided a useful model for characterizing and annotating rat noncoding genes, covering multi-organs and various development periods in both sexes. To the best of our knowledge, it is the first study to build a comprehensive and reliable platform for monitoring the expression fluctuation of noncoding genes among multiple organs along with aging, which builds the foundation for future research on noncoding gene-expression profiles in rat. Moreover, our results can facilitate understanding on changes of noncoding genes expression during developmental stages, providing us an opportunity to assess the potential functions of ncRNAs associated with each condition during the development of organs. Our work will benefit biomedical research as well as drug development by facilitating comprehensive expression profiling and functional annotation of noncoding genes in rat.

## Methods

### Raw reads alignment and expression quantification

The raw reads were downloaded from Ying *et al*.’s deposit in GEO (GSE53960). The data set consisted of transcriptome sequencing data (50 bp single end and ribosomal removal protocol) of 320 rat bodymap samples from 32 rats with 10 organs (adrenal gland, brain, heart, kidney, liver, lung, skeletal muscle, spleen, thymus, and testis or uterus) from each rat. The 32 rats were sacrificed at four different ages (i.e. 2 weeks, 6 weeks, 21 weeks and 104 weeks old) for both sexes (male and female) in four biological replicates.

Raw reads were mapped back to the rat reference genome Ensembl^49^ Rnor_5.0 by TopHat2 v2.0.13^50^, followed by Cufflinks v2.2.1, for gene assembly and quantification. FPKM (Fragment Per Kilobase per Million mapped reads) was used for reflecting the expression level. To avoid infinite values, we added 0.05 to each FPKM value before log 2 transformation. A gene is defined as expressed when its FPKM is greater than 0.01.

### Selection of noncoding genes and analysis of expression profiles

As we have four biological replicates for each of the 80 different conditions, Cuffnorm was performed among 320 samples to access the significant changes in expression for genes between any two conditions. After the expression level was normalized for each gene, we excluded the protein-coding genes from the expression matrix and 3,458 expressed noncoding genes remained. We applied hierarchical clustering analysis (HCA) to visualize the overall expression profiles. Principal variance component analysis (PVCA) with R^51^ was used to study the relative contribution to the total variance from organ, age, sex, and replicate.

### Identification of differentially expressed noncoding genes

With the aligned results by TopHat2, we used Cuffdiff, a downstream program within Cufflinks, together with the rat Ensembl Rnor_5.0 GTF file, to conduct noncoding transcriptome assembly and to identify differentially expressed noncoding genes (DEnGs) related to organ, age and sex. The threshold of false discovery rate (FDR) adjusted *p*-value was set as 0.05 for differential expression analysis. For organ dependent DEnGs analysis, we combined male and female rats together in each time point and compared each pair of two different organs (adjusted *p* ≤ 0.05). Age-related DEnGs were selected by the same method as that of organ-related DEnGs. We only compared the adjacent age points in a time series, and sexes were considered separately. Similarly, sex-related DEnGs were examined across the four developmental stages among ten distinct organs.

### Gene selection for co-expression network construction and functional enrichment analysis

We first excluded those lowly expressed genes with FPKM < 0.1. Then, we performed an analysis of variance (ANOVA) on all 26,689 rat genes and used Benjamini-Hochberg adjusted *p* ≤ 0.05 to select genes that were potentially functionally and specifically relevant to organ, age and sex. Finally, 16,346 genes were identified as organ-related, 3,572 genes as age-related, and 1,603 genes as sex-related. The weighted gene co-expression network analysis between protein-coding genes and noncoding genes was constructed using WGCNA package^37^. We performed co-expression analysis separately in terms of organ, age and sex so as to explore the influence induced by the connection and interactions between protein-coding genes and noncoding genes. The choice of power (β) should be the smallest value to make sure that the approximate scale free topology is reached. In our study, we raised the power to 6, 8 and 7 while analyzing on organ, age and sex respectively, producing a weighted correlation matrix, representing the interactions of genes. After turning it into an adjacency matrix, we performed hierarchical clustering to group genes based on topological overlap dissimilarity to indicate their real connection strengths. Genes grouped in the same module were similarly expressed in expression patterns and shared a similar biological function or became a part of the same biological pathway. The functional enrichment analysis based on each interested module was finished within DAVID^52^ by including both gene ontology (GO) and the Kyoto encyclopedia of genes and genomes (KEGG) pathways.

### Visualization of the eigengene network among modules with age

Two selection standards were set to meet the criteria. First, we chose modules with more noncoding genes so that their possible biological roles can be inferred by those of the protein-coding genes involved in the same pathway or biological process. Secondly, we set a threshold for each module to control the strength of inter-nodes connectivity. By referring to the eigengene dendrogram that reveals the relationships between modules and factors, one can choose modules that are relatively highly related to a trait (i.e. age). One must make sure that their mutual correlations were stronger than other modules to age, for instance. Finally, we exported the edge file of those genes into Cytoscape v3.2.1 to make a network so as to specify the weighted link among genes.

## Additional Information

**How to cite this article**: Wen, Z. *et al*. Expression profiling and functional annotation of noncoding genes across 11 distinct organs in rat development. *Sci. Rep.* **6**, 38575; doi: 10.1038/srep38575 (2016).

**Publisher's note:** Springer Nature remains neutral with regard to jurisdictional claims in published maps and institutional affiliations.

## Supplementary Material

Supplementary Information
