# Supplementary material for: Expression profiling and functional annotation of noncoding genes across 11 distinct organs in rat development
Source: Sci Rep. 2016 Dec 9;6:38575. doi: 10.1038/srep38575 (PMC5146941; doi:10.1038/srep38575)
Supplement: Supplementary Information [file srep38575-s1.pdf]

Expression profiling and functional annotation of noncoding genes across 11 distinct organs in rat development

Zhuo Wen[1,2], Geng Chen[2], Sibo Zhu[2,3], Jinhang Zhu[2], Bin Li[2], Yunjie Song[2], Suqing Li[1], Leming Shi[2,3] , Yuanting Zheng[2,3*] & Menglong Li[1*]

[1]College of Chemistry, Sichuan University, Chengdu 610064, China.

[2]Center for Pharmacogenomics, School of Pharmacy, and State Key Laboratory of Genetic Engineering and MOE Key Laboratory of Contemporary Anthropology, School of Life Sciences, Fudan University, Shanghai 201203, China.

[3]Collaborative Innovation Center for Genetics and Development, Fudan University, Shanghai 200438, China.

*To whom correspondence should be addressed (zhengyuanting@fudan.edu.cn and liml@scu.edu.cn).

Supplementary tables

S1 Enrichment analysis of organ-related modules (top20)

Module	Order	GO ID	GO Term	P value
blue	1	GO:0019226	transmission of nerve impulse	2.00E-45
blue	2	GO:0007268	synaptic transmission	2.10E-44
blue	3	GO:0007267	cell-cell signaling	4.40E-39
blue	4	GO:0051969	regulation of transmission of nerve impulse	2.60E-30
blue	5	GO:0031644	regulation of neurological system process	3.90E-29
blue	6	GO:0050804	regulation of synaptic transmission	1.10E-28
blue	7	GO:0006836	neurotransmitter transport	9.50E-26
blue	8	GO:0006811	ion transport	5.10E-22
blue	9	GO:0030182	neuron differentiation	5.30E-20
blue	10	GO:0007610	behavior	1.10E-19
blue	11	GO:0007269	neurotransmitter secretion	7.10E-18
blue	12	GO:0001505	regulation of neurotransmitter levels	1.90E-17
blue	13	GO:0044057	regulation of system process	3.70E-17
blue	14	GO:0048666	neuron development	6.00E-15
blue	15	GO:0006812	cation transport	1.00E-14
blue	16	GO:0030001	metal ion transport	2.60E-14
blue	17	GO:0048489	synaptic vesicle transport	2.90E-14
blue	18	GO:0031175	neuron projection development	6.60E-14
blue	19	GO:0007611	learning or memory	2.20E-13
blue	20	GO:0046903	secretion	2.90E-13
darkturquoise	1	GO:0006955	immune response	1.70E-41
darkturquoise	2	GO:0002684	positive regulation of immune system process	2.00E-36

darkturquoise	3	GO:0045321	leukocyte activation	1.60E-31
darkturquoise	4	GO:0001775	cell activation	1.90E-31
darkturquoise	5	GO:0050865	regulation of cell activation	2.80E-31
darkturquoise	6	GO:0002694	regulation of leukocyte activation	2.10E-29
darkturquoise	7	GO:0051249	regulation of lymphocyte activation	4.30E-27
darkturquoise	8	GO:0006952	defense response	2.60E-25
darkturquoise	9	GO:0046649	lymphocyte activation	1.10E-23
darkturquoise	10	GO:0050778	positive regulation of immune response	1.10E-22
darkturquoise	11	GO:0048584	positive regulation of response to stimulus	1.10E-21
darkturquoise	12	GO:0050867	positive regulation of cell activation	1.50E-20
darkturquoise	13	GO:0050863	regulation of T cell activation	2.80E-19
darkturquoise	14	GO:0001817	regulation of cytokine production	3.80E-19
darkturquoise	15	GO:0050670	regulation of lymphocyte proliferation	4.30E-19
darkturquoise	16	GO:0032944	regulation of mononuclear cell proliferation	6.30E-19
darkturquoise	17	GO:0070663	regulation of leukocyte proliferation	1.40E-18
darkturquoise	18	GO:0002696	positive regulation of leukocyte activation	3.10E-18
darkturquoise	19	GO:0002252	immune effector process	4.90E-18
darkturquoise	20	GO:0051251	positive regulation of lymphocyte activation	1.10E-16
turquoise	1	GO:0019953	sexual reproduction	1.30E-21
turquoise	2	GO:0048232	spermatogenesis	8.40E-19
turquoise	3	GO:0007283	male gamete generation	8.40E-19
turquoise	4	GO:0007276	gamete generation	3.60E-18

turquoise	5	GO:0032504	multicellular organism reproduction	7.30E-15
turquoise	6	GO:0048609	reproductive process in a multicellular organism	7.30E-15
turquoise	7	GO:0048610	reproductive cellular process	1.40E-10
turquoise	8	GO:0007286	spermatid development	2.60E-10
turquoise	9	GO:0048515	spermatid differentiation	6.60E-10
turquoise	10	GO:0007281	germ cell development	7.50E-10
turquoise	11	GO:0034470	ncRNA processing	9.90E-07
turquoise	12	GO:0034660	ncRNA metabolic process	1.50E-06
turquoise	13	GO:0009566	fertilization	1.80E-06
turquoise	14	GO:0006396	RNA processing	1.80E-05
turquoise	15	GO:0008033	tRNA processing	5.80E-05
turquoise	16	GO:0007338	single fertilization	6.60E-05
turquoise	17	GO:0006350	transcription	9.30E-05
turquoise	18	GO:0003006	reproductive developmental process	3.00E-04
turquoise	19	GO:0006508	proteolysis	3.90E-04
turquoise	20	GO:0044265	cellular macromolecule catabolic process	8.20E-04
green	1	GO:0006259	DNA metabolic process	1.70E-48
green	2	GO:0007049	cell cycle	1.00E-46
green	3	GO:0022402	cell cycle process	3.60E-46
green	4	GO:0022403	cell cycle phase	4.10E-44
green	5	GO:0000279	M phase	9.30E-42
green	6	GO:0006260	DNA replication	8.40E-41
green	7	GO:0000087	M phase of mitotic cell cycle	5.50E-30
green	8	GO:0000278	mitotic cell cycle	6.20E-30
green	9	GO:0007067	mitosis	1.50E-28
green	10	GO:0000280	nuclear division	1.50E-28

green	11	GO:0006281	DNA repair	5.90E-28
green	12	GO:0048285	organelle fission	2.60E-27
green	13	GO:0006974	response to DNA damage stimulus	4.70E-27
green	14	GO:0051276	chromosome organization	2.00E-24
green	15	GO:0051301	cell division	8.90E-23
green	16	GO:0007059	chromosome segregation	3.00E-22
green	17	GO:0006396	RNA processing	5.60E-22
green	18	GO:0016071	mRNA metabolic process	1.20E-20
green	19	GO:0033554	cellular response to stress	1.40E-19
green	20	GO:0051726	regulation of cell cycle	2.00E-17
lightgreen	1	GO:0006091	generation of precursor metabolites and energy	8.50E-46
lightgreen	2	GO:0045333	cellular respiration	1.20E-44
lightgreen	3	GO:0022900	electron transport chain	1.00E-33
lightgreen	4	GO:0015980	energy derivation by oxidation of organic compounds	2.50E-33
lightgreen	5	GO:0006084	acetyl-CoA metabolic process	1.00E-25
lightgreen	6	GO:0051186	cofactor metabolic process	1.20E-24
lightgreen	7	GO:0055114	oxidation reduction	1.70E-24
lightgreen	8	GO:0022904	respiratory electron transport chain	2.40E-24
lightgreen	9	GO:0006119	oxidative phosphorylation	2.90E-22
lightgreen	10	GO:0007005	mitochondrion organization	5.20E-22
lightgreen	11	GO:0006732	coenzyme metabolic process	1.60E-21
lightgreen	12	GO:0009060	aerobic respiration	3.60E-21
lightgreen	13	GO:0051188	cofactor biosynthetic process	2.30E-13
lightgreen	14	GO:0006631	fatty acid metabolic process	1.60E-10
lightgreen	15	GO:0055085	transmembrane transport	9.50E-10
lightgreen	16	GO:0046395	carboxylic acid catabolic process	1.30E-09

lightgreen	17	GO:0016054	organic acid catabolic process	1.30E-09
lightgreen	18	GO:0016042	lipid catabolic process	3.50E-08
lightgreen	19	GO:0006412	translation	2.10E-07
lightgreen	20	GO:0032269	negative regulation of cellular protein metabolic process	2.30E-06
black	1	GO:0030001	metal ion transport	4.50E-04
black	2	GO:0005996	monosaccharide metabolic process	4.50E-04
black	3	GO:0006812	cation transport	9.60E-04
black	4	GO:0006813	potassium ion transport	9.80E-04
black	5	GO:0009628	response to abiotic stimulus	1.00E-03
black	6	GO:0051051	negative regulation of transport	3.20E-03
black	7	GO:0015672	monovalent inorganic cation transport	4.10E-03
black	8	GO:0032535	regulation of cellular component size	4.30E-03
black	9	GO:0019318	hexose metabolic process	4.50E-03
black	10	GO:0006811	ion transport	5.00E-03
black	11	GO:0008361	regulation of cell size	6.70E-03
black	12	GO:0044057	regulation of system process	9.20E-03
black	13	GO:0006006	glucose metabolic process	1.40E-02
black	14	GO:0009725	response to hormone stimulus	1.40E-02
black	15	GO:0051347	positive regulation of transferase activity	1.50E-02
black	16	GO:0045860	positive regulation of protein kinase activity	2.20E-02
black	17	GO:0009719	response to endogenous stimulus	2.30E-02
black	18	GO:0016477	cell migration	2.40E-02
black	19	GO:0051674	localization of cell	2.50E-02
black	20	GO:0048870	cell motility	2.50E-02

S2 Enrichment analysis of sex-related modules (top10)

Module	Order	GO ID	GO Term	P-Value
turquoise	1	GO:0019953	sexual reproduction	4.80E-34
turquoise	2	GO:0007283	male gamete generation	1.20E-29
turquoise	3	GO:0048232	spermatogenesis	1.20E-29
turquoise	4	GO:0007276	gamete generation	5.90E-28
turquoise	5	GO:0048609	multicellular organism reproduction	3.20E-25
turquoise	6	GO:0032504	reproductive process in a multicellular organism	3.20E-25
turquoise	7	GO:0048610	reproductive cellular process	1.00E-14
turquoise	8	GO:0007286	spermatid development	1.10E-11
turquoise	9	GO:0048515	spermatid differentiation	2.50E-11
turquoise	10	GO:0007281	germ cell development	6.90E-10
brown	1	GO:0007049	cell cycle	3.20E-07
brown	2	GO:0000279	M phase	2.00E-06
brown	3	GO:0022402	cell cycle process	2.80E-06
brown	4	GO:0022403	cell cycle phase	1.40E-05
brown	5	GO:0006259	DNA metabolic process	2.40E-05
brown	6	GO:0051276	chromosome organization	1.00E-03
brown	7	GO:0051726	regulation of cell cycle	1.60E-03
brown	8	GO:0006281	DNA repair	3.70E-03
brown	9	GO:0000278	mitotic cell cycle	7.50E-03
brown	10	GO:0001701	in utero embryonic development	8.50E-03

S3 Enrichment analysis of age-related modules (top20)

Module	Order	GO ID	GO Term	P value
blue	1	GO:0022402	cell cycle process	7.00E-33
blue	2	GO:0007049	cell cycle	9.70E-32
blue	3	GO:0000279	M phase	1.20E-31
blue	4	GO:0022403	cell cycle phase	4.30E-30
blue	5	GO:0000087	M phase of mitotic cell cycle	6.10E-30
blue	6	GO:0000280	nuclear division	2.30E-28
blue	7	GO:0007067	mitosis	2.30E-28
blue	8	GO:0048285	organelle fission	3.20E-27
blue	9	GO:0006259	DNA metabolic process	9.30E-27
blue	10	GO:0000278	mitotic cell cycle	1.60E-25
blue	11	GO:0007059	chromosome segregation	4.00E-23
blue	12	GO:0006260	DNA replication	3.40E-21
blue	13	GO:0051301	cell division	1.80E-19
blue	14	GO:0051276	chromosome organization	1.20E-17
blue	15	GO:0006281	DNA repair	9.70E-17
blue	16	GO:0006974	response to DNA damage stimulus	1.90E-16
blue	17	GO:0016071	mRNA metabolic process	1.60E-14
blue	18	GO:0000377	RNA splicing, via transesterification reactions	4.50E-14
blue	19	GO:0000375	nuclear mRNA splicing, via spliceosome	4.50E-14
blue	20	GO:0000398	RNA splicing, via transesterification reactions with bulged adenosine as nucleophile	4.50E-14
brown	1	GO:0006955	immune response	2.40E-44
brown	2	GO:0006952	defense response	2.50E-25
brown	3	GO:0002684	positive regulation of immune system process	2.10E-24

brown	4	GO:0045321	leukocyte activation	3.10E-21
brown	5	GO:0050778	positive regulation of immune response	8.30E-21
brown	6	GO:0001775	cell activation	5.20E-20
brown	7	GO:0048584	positive regulation of response to stimulus	7.90E-20
brown	8	GO:0002252	immune effector process	1.10E-17
brown	9	GO:0050865	regulation of cell activation	1.80E-16
brown	10	GO:0001817	regulation of cytokine production	1.30E-15
brown	11	GO:0046649	lymphocyte activation	1.70E-15
brown	12	GO:0002694	regulation of leukocyte activation	3.90E-15
brown	13	GO:0002443	leukocyte mediated immunity	3.20E-14
brown	14	GO:0042110	T cell activation	1.60E-13
brown	15	GO:0006954	inflammatory response	3.10E-13
brown	16	GO:0001819	positive regulation of cytokine production	3.90E-13
brown	17	GO:0051249	regulation of lymphocyte activation	7.80E-13
brown	18	GO:0050867	positive regulation of cell activation	9.80E-13
brown	19	GO:0002253	activation of immune response	1.30E-12
brown	20	GO:0002697	regulation of immune effector process	7.00E-12
